# Do age-related macular degeneration genes show association with keratoconus?

**DOI:** 10.1186/s40662-019-0164-z

**Published:** 2019-12-01

**Authors:** Ke Cao, Srujana Sahebjada, Andrea J. Richardson, Paul N. Baird

**Affiliations:** 1 Centre for Eye Research Australia, Royal Victorian Eye and Ear Hospital, Melbourne, Australia; 2 Department of Surgery, Ophthalmology, The University of Melbourne, 32 Gisborne Street, East Melbourne, VIC 3000 Australia

**Keywords:** Keratoconus, Gender, Genetic, Age-related macular degeneration

## Abstract

**Background:**

Keratoconus (KC) is a common corneal condition with an unknown gender predominance. Although numerous studies have investigated the genetic component of KC, no specific genes have yet been attributed to the condition. We recently reported posterior segment changes occurring in the eyes of KC patients. However, it is not clear whether these changes are part of KC pathogenesis or reflect changes in anatomical features of the eye manifested by changes at the cornea. Given retinal changes represent the main characteristics observed in age-related macular degeneration (AMD) and that pleiotropy has been demonstrated between different eye diseases, we wished to assess if known AMD associated genes were also associated with KC.

**Methods:**

A total of 248 KC subjects and 366 non-KC (control) subjects were recruited from public and private clinics in Melbourne for this analysis. Nineteen single nucleotide polymorphisms (SNPs) previously associated with AMD, including rs10490924 (*ARMS2/HTRA1*), rs10737680 (*CFH*), rs13278062 (*TNFRSF10A*), rs1864163 (*CETP*), rs2230199 (*C3*), rs3130783 (*IER3/DDR1*), rs334353 (*TGFBR1*), rs3812111 (*COL10A1*), rs429608 (*C2/CFB*), rs4420638 (*APOE*), rs4698775 (*CFI*), rs5749482 (*TIMP3*), rs6795735 (*ADAMTS9*), rs8017304 (*RAD51B*), rs8135665 (*SLC16A8*), rs920915 (*LIPC*), rs943080 (*VEGFA*), rs9542236 (*B3GALTL*) and rs13081855 (*COL8A1/FILIP1L*), were genotyped in this cohort. Logistic regression was applied to evaluate the association between these SNPs and KC on both genders together, as well as each gender separately. Linear regression was also applied to assess the association between SNPs and corneal curvature. Bonferroni correction was applied to adjust for multiple testing.

**Results:**

Genotyping data were available for 18 SNPs. The SNP, rs6795735 (*ADAMTS9*) was significantly associated with KC (*p* = 3.5 × 10^− 4^) when both genders were assessed, whereas rs5749482 (*TIMP3*) was only associated in males (*p* = 7.7 × 10^− 4^) following Bonferroni multiple correction. However, when the covariates of age and gender were included, the associations became non-significant. In addition, none of the SNPs appeared significant for corneal curvature.

**Conclusions:**

Our study suggested a potential association of rs6795735 in the *ADAMTS9* gene and rs5749482 in the *TIMP3* gene in KC and that different associations may be gender specific. Overall, SNPs initially identified as associated with AMD following multiple correction may be further impacted by other factors such as age or gender and further studies are needed to resolve this issue.

## Background

Keratoconus (KC) is a progressive, bilateral and asymmetric corneal condition characterised by corneal thinning which leads to significant visual impairment [1] and accounted for 31% of all corneal grafts in Australia in 2018 [2]. Although spectacles, contact lens or other optical interventions can often correct some, or all visual acuity, they have no role in preventing its progression [3]. Corneal collagen cross-linking (CXL) has been shown to slow KC progression by increasing the stiffness of the cornea, but this can only be done at the early stage of disease when there is maximal corneal thickness [4].

The prevalence of KC appears to be increasing around the world with an often quoted US study published in 1982 reporting a prevalence of KC of 1:2000 [5]. However, a more recent study in 2017 based on findings from a Dutch health provider reported a KC prevalence of 1:375 [6]. This rapid and large increase in prevalence most likely reflects our increased ability to detect early changes in KC through availability of advanced and highly sensitive anterior segment imaging techniques. KC affects both genders and while a number of studies have indicated a higher preponderance of males with KC there are still conflicting results on gender predominance [1, 7]. The Collaborative Longitudinal Evaluation of Keratoconus (CLEK) Study investigated 1209 KC patients in the United States and reported a male/female ratio of 1.33 [8]. Godefrooij et al. in the Netherlands and Woodward et al. in the US also reported a higher percentage of males in their KC patients, with male/female ratio of 1.54 and 1.43 respectively [6, 9]. Conversely, the studies by Jonas et al. and Hashemi et al. reported opposite results indicating more females with a male/female ratio of 0.29 and 0.58 in India and Tehran, respectively [10, 11]. In addition, gender differences exist in family history, clinical parameters, symptoms and treatment prognosis in KC patients [12, 13].

KC is a complex multifactorial condition with both genetic and environmental factors playing a role in its aetiology [14]. Recent studies have made efforts to identify genetic risk factors for KC, but only a small portion of the overall genetic components have been identified. Genetic linkage studies have reported in at least 17 gene loci, indicating the likely presence of multiple genes involved in KC [15]. However, the identification of true disease-causing genes has been scarce. For the many candidate genes reported to be associated with KC, few of the early detected genes have been replicated [16]. More recently, a genome-wide association study (GWAS) that included samples from Australia, the USA and Northern Ireland identified a putative association of the Hepatocyte growth factor (*HGF*) gene which, although falling short of genome-wide significance was independently replicated by us [17, 18]. Further studies to identify causative genes associated with KC are therefore needed.

One way of identifying causative KC genes may be through the analysis of genes identified through other complex eye diseases. For example, central corneal thickness (CCT) and increased corneal curvature represent the two main continuous traits observed in KC. Lu et al. [19] were able to demonstrate that of the 27 CCT genes identified through a GWAS for glaucoma, six of these were also associated with CCT in KC. We were able to subsequently replicate 2 of these genes – the *MPDZ-NF1B* gene and the *BANP/ZNF4659* gene locus in an independent KC study [20].

In light of the finding that genetic associations identified in one complex eye disease could also be pleiotropic with KC, we further investigated our previous observation of structural posterior segment changes in the eyes of KC patients [21]. These changes were detected by optical coherence tomography (OCT) and indicated a significantly greater mean retinal thickness in the central fovea, inner and outer macula and increased macular volume compared to non-keratoconus patients. It is currently unknown whether these changes occur as part of KC disease aetiology or reflect a change in eye dimensions resulting from corneal curvature changes at the anterior segment. Retinal changes also represent a key characteristic in the complex disease age-related macular degeneration (AMD), albeit it from a thinning of the retina. A number of genetic associations with AMD have been identified by the International AMD Gene Consortium in two previous reports [22, 23]. We therefore undertook a case-control genetic association study to investigate the top 19 single nucleotide polymorphisms (SNPs) previously associated with AMD loci reported by Fritsche et al. (2013) to assess their associations with KC on both genders as well as each gender separately, as well as their association with corneal curvature [23].

## Methods

The study protocol was approved by the Royal Victorian Eye and Ear Hospital (RVEEH) Human Research and Ethics Committee (Project#10/954H). This protocol followed the tenets of the Declaration of Helsinki and all privacy requirements were met.

KC patients with a European background were recruited from public and private clinics at the RVEEH, private rooms, optometry clinics or consenting general public with KC. A comprehensive eye examination was undertaken for each patient and the diagnostic criteria for KC has been described in detail elsewhere [18]. Briefly, KC was diagnosed on the basis of the presence of one or more of the following: [1] an irregular cornea, as determined by distortion of keratometric mires and/or orbscan/pentacam images, [2] scissoring of the retinoscopic reflex; and [3] demonstration of at least one bio microscopic sign, including Vogt’s striae, Fleischer’s ring or corneal thinning and scarring typical of KC. Potential subjects with non-KC ocular disease in both eyes such as keratectasia, corneal degenerations, macular disease, and optic nerve disease (e.g., optic neuritis, optic atrophy) were excluded from the study.

Non-KC subjects (controls) were recruited from the ‘GEnes in Myopia (GEM)’ study, where a similar testing protocol was used and has been previously described [24]. Individuals in the GEM study were excluded if they had known ocular disease or insult that could predispose to myopia such as KC. A blood or saliva sample was collected from each subject for subsequent genetic analysis.

### SNP selection and genotyping

Deoxyribonucleic acid (DNA) was extracted from blood or saliva samples using NucleoSpin® QuickPure kits and genotyping was performed through the Mass Array platform (Agena Bioscience, San Diego, CA) at the Murdoch Children’s Research Institute, Melbourne.

A total of 19 SNPs previously associated with AMD were genotyped. These included rs10490924 (*ARMS2/HTRA1*), rs10737680 (*CFH*), rs13278062 (*TNFRSF10A*), rs1864163 (*CETP*), rs2230199 (*C3*), rs3130783 (*IER3/DDR1*), rs334353 (*TGFBR1*), rs3812111 (*COL10A1*), rs429608 (*C2/CFB*), rs4420638 (*APOE*), rs4698775 (*CFI*), rs5749482 (*TIMP3*), rs6795735 (*ADAMTS9*), rs8017304 (*RAD51B*), rs8135665 (*SLC16A8*), rs920915 (*LIPC*), rs943080 (*VEGFA*), rs9542236 (*B3GALTL*) and rs13081855 (*COL8A1/FILIP1L*) (Additional file 1: Table S1). The SNP rs13081855 was excluded from our analysis due to a low genotyping quality. For each SNP, alleles, genotypes, odd ratio (OR) and 95% confidence intervals (95% CI) were established.

### Statistical analysis

Data were first analysed with RStudio (Version 1.1.456) for Windows. All statistical tests were considered significant when the *p*-value was less than 0.05. A student’s t-test was used to compare age between groups, and a Wilcoxon signed-rank test was applied to test the difference of other clinical characteristics, including corneal curvature, spherical equivalent, axial length and anterior chamber depth.

PLINK v1.07 (http://zzz.bwh.harvard.edu/plink/download.shtml) was applied to perform the following analyses:

1. Logistic regression for testing case/control association with and without covariates (age and gender) adjustment, performed on both genders as well as male and female genders separately;
2. Linear regression for quantitative trait analysis of the selected SNPs for corneal curvature with and without covariate (age) adjustment.

All patients had bilateral eye data, and so only data on right eyes were used for analysis. Bonferroni correction was used to adjust for multiple tests to a level of *p* < 0.05.

Power calculations were performed using the online statistical calculator (http://osse.bii.a-star.edu.sg/calculation2.php) with an alpha of 0.05 using a case-control design, based on the minor allele frequency (MAF).

## Results

In total, 614 subjects consisting 248 KC subjects and 366 non-KC subjects were available for analysis. Age and gender were available for all individuals with there being 96 females (38.7%) in the KC and 232 females (63.4%) in the non-KC group. The mean age of KC patients was 35.6 ± 14.8 years and non-KC was 48.4 ± 13.5 years. The mean age in males and females in KC was 33.3 ± 13.5 and 39.1 ± 16.0 years, respectively and in non- KC (controls) was 49.3 ± 13.3 and 47.9 ± 13.6 years, respectively. KC patients were significantly younger compared with the non-KC group (*p* < 0.01) and this was the case for both males and females. Demographics for both KC and non-KC are shown (Table 1).

**Table 1:** Demographics for each group

	*n*	Mean age (years) (SD)	% Female
KC	248	35.6 (14.8)	38.7
Non-KC	366	48.4 (13.5)	63.4

*KC* = keratoconus, *SD* = standard deviation

Mean corneal curvature was available for 547 subjects of which 226 out of 248 (91.1%) were available for the KC group and 321 out of 366 (87.7%) were available for the non-KC group. KC patients had a steeper cornea than the non-KC group (*p* < 0.01). Spherical equivalent was available for 517 subjects of which 158 out of 248 (63.7%) were available for the KC group and 359 out of 366 (98.1%) were available for the non-KC group. KC subjects tended to be more myopic (*p* < 0.01). There was no significant difference in axial length and anterior chamber depth between groups. Clinical characteristics for each group are shown (Table 2).

**Table 2:** Clinical characteristics for each group

	KC subjects	Non-KC subjects
Corneal curvature (D) (SD)	49.46 (7.97)	42.34 (2.68)
Spherical equivalent (D) (SD)	−4.80 (5.22)	−2.63 (3.42)
Axial length (mm) (SD)	24.38 (1.62)	24.65 (1.53)
Anterior chamber depth (mm) (SD)	3.53 (0.58)	3.50 (0.39)

*KC* = keratoconus, *D* = Dioptres, *SD* = standard deviation

A total of 18 SNPs were included in the analysis including rs10490924 (ARMS2/HTRA1), rs10737680 (CFH), rs13278062 (TNFRSF10A), rs1864163 (CETP), rs2230199 (C3), rs3130783 (IER3/DDR1), rs334353 (TGFBR1), rs3812111 (COL10A1), rs429608 (C2/CFB), rs4420638 (APOE), rs4698775 (CFI), rs5749482 (TIMP3), rs6795735 (ADAMTS9), rs8017304 (RAD51B), rs8135665 (SLC16A8), rs920915 (LIPC), rs943080 (VEGFA) and rs9542236 (B3GALTL).

Genetic association was performed using logistic regression to evaluate the association of the 18 SNPs with KC. For each SNP, the corrected Bonferroni adjusted *P* value of 0.05/18 = 2.8 × 10^− 3^ was considered statistically significant. Of the 18 SNPs, when considering both genders, only SNP rs6795735 (ADAMTS9) showed a significant association (*p* = 3.5 × 10^− 4^) (Table 3). However, the SNP rs5749482 (TIMP3) also showed significant association (*p* = 7.7 × 10^− 4^) with KC when only males were considered in the analysis (Table 4). There was no significant association for females. Following the inclusion of age as a covariate (as age was significantly different between KC and non-KC groups), the associations became non-significant (Tables 3 & 4).

**Table 3:** Logistic regression analysis for the assessment of AMD associated genes with KC in both genders

			Without adjusting for age & gender		Adjusting for age & gender	
CHR	SNP	Minor Allele Name	Odds Ratio (95% CI)	*P*	Odds Ratio (95% CI)	*P*
3	rs6795735	T	1.52 (1.21–1.92)	**3.5 × 10**^**−4**^	1.35 (1.04–1.75)	2.2 × 10^−2^
22	rs5749482	C	1.53 (1.13–2.09)	6.6 × 10^− 3^	1.28 (0.90–1.82)	1.7 × 10^− 1^
13	rs9542236	C	0.73 (0.58–0.92)	7.8 × 10^− 3^	0.82 (0.64–1.07)	1.4 × 10^− 1^
10	rs10490924	T	1.34 (1.03–1.76)	3.1 × 10^−2^	1.27 (0.94–1.72)	1.2 × 10^− 1^
6	rs943080	C	0.79 (0.63–0.99)	4.0 × 10^−2^	0.91 (0.70–1.17)	4.4 × 10^− 1^
19	rs2230199	G	0.79 (0.58–1.07)	1.2 × 10^− 1^	0.77 (0.55–1.09)	1.4 × 10^− 1^
6	rs429608	A	1.25 (0.92–1.70)	1.5 × 10^− 1^	1.33 (0.94–1.88)	1.1 × 10^− 1^
16	rs1864163	A	0.82 (0.63–1.08)	1.5 × 10^−1^	0.95 (0.70–1.28)	7.1 × 10^− 1^
4	rs4698775	G	1.20 (0.93–1.55)	1.6 × 10^−1^	1.27 (0.95–1.69)	1.0 × 10^− 1^
15	rs920915	C	0.85 (0.68–1.07)	1.7 × 10^−1^	0.89 (0.69–1.16)	3.8 × 10^− 1^
1	rs10737680	C	1.14 (0.91–1.43)	2.6 × 10^−1^	1.02 (0.79–1.32)	8.7 × 10^− 1^
6	rs3812111	A	0.88 (0.70–1.11)	2.7 × 10^−1^	0.82 (0.63–1.06)	1.3 × 10^− 1^
8	rs13278062	G	0.90 (0.72–1.13)	3.7 × 10^−1^	0.89 (0.70–1.15)	3.8 × 10^− 1^
14	rs8017304	G	1.11 (0.88–1.39)	3.9 × 10^−1^	1.06 (0.82–1.37)	6.5 × 10^− 1^
6	rs3130783	G	0.90 (0.68–1.19)	4.7 × 10^−1^	0.93 (0.68–1.26)	6.3 × 10^− 1^
19	rs4420638	G	0.91 (0.68–1.21)	5.1 × 10^−1^	0.83 (0.60–1.16)	2.7 × 10^− 1^
22	rs8135665	A	1.08 (0.81–1.45)	6.0 × 10^−1^	1.28 (0.92–1.79)	1.4 × 10^− 1^
9	rs334353	G	0.97 (0.75–1.25)	8.0 × 10^−1^	1.00 (0.75–1.34)	9.8 × 10^− 1^

*AMD* = age-related macular degeneration, *KC* = keratoconus, *CI* = confidence intervals, *CHR* = chromosome, *SNP* = single nucleotide polymorphisms

*P* value for statistical significance is 0.05/18  =  2.8 × 10^−3^

Bolded value indicates significance

95% CI – 95% confidence interval

**Table 4:** Logistic regression analysis for the assessment of AMD associated genes with KC in males

			Without age adjustment		Adjusting for age	
CHR	SNP	Minor Allele Name	Odds Ratio (95% CI)	*P*	Odds Ratio (95% CI)	P
22	rs5749482	C	2.29 (1.41–3.71)	**7.7 × 10**^**−4**^	1.76 (1.01–3.08)	4.8 × 10^−2^
3	rs6795735	T	1.60 (1.15–2.23)	5.4 × 10^− 3^	1.36 (0.93–1.99)	1.1 × 10^− 1^
6	rs943080	C	0.68 (0.50–0.94)	1.9 × 10^−2^	0.75 (0.52–1.09)	1.4 × 10^− 1^
13	rs9542236	C	0.72 (0.52–1.00)	4.8 × 10^−2^	0.97 (0.66–1.42)	8.7 × 10^− 1^
10	rs10490924	T	1.44 (0.99–2.10)	5.7 × 10^−2^	1.54 (0.99–2.40)	5.4 × 10^− 2^
6	rs429608	A	1.49 (0.95–2.34)	7.9 × 10^−2^	1.77 (1.05–2.99)	3.1 × 10^− 2^
15	rs920915	C	0.75 (0.54–1.05)	8.9 × 10^−2^	0.82 (0.56–1.21)	3.2 × 10^−1^
14	rs8017304	G	1.31 (0.93–1.85)	1.2 × 10^−1^	1.31 (0.88–1.96)	1.9 × 10^−1^
9	rs334353	G	0.85 (0.59–1.23)	4.0 × 10^−1^	0.91 (0.59–1.39)	6.6 × 10^− 1^
8	rs13278062	G	0.88 (0.64–1.23)	4.6 × 10^−1^	0.85 (0.58–1.25)	4.1 × 10^−1^
16	rs1864163	A	0.86 (0.57–1.29)	4.6 × 10^−1^	0.92 (0.58–1.47)	7.4 × 10^− 1^
6	rs3812111	A	0.89 (0.64–1.24)	4.9 × 10^−1^	0.81 (0.55–1.19)	2.8 × 10^−1^
19	rs2230199	G	0.88 (0.58–1.33)	5.4 × 10^−1^	1.05 (0.65–1.71)	8.3 × 10^− 1^
22	rs8135665	A	1.09 (0.73–1.63)	6.6 × 10^−1^	1.35 (0.85–2.17)	2.1 × 10^−1^
4	rs4698775	G	0.96 (0.67–1.37)	8.2 × 10^−1^	1.05 (0.69–1.60)	8.3 × 10^− 1^
19	rs4420638	G	1.05 (0.70–1.57)	8.3 × 10^−1^	0.90 (0.56–1.46)	6.8 × 10^−1^
6	rs3130783	G	0.98 (0.66–1.45)	9.1 × 10^−1^	1.10 (0.69–1.75)	7.0 × 10^− 1^
1	rs10737680	C	1.01 (0.72–1.42)	9.6 × 10^−1^	0.78 (0.52–1.17)	2.2 × 10^−1^

*AMD* = age-related macular degeneration, *KC* = keratoconus, *CHR* = chromosome, *CI* = confidence intervals, *SNP* = single nucleotide polymorphisms

*P* value for statistical significance is 0.05/18  = 2.8 × 10^−3^

Bolded value indicates significance

95% CI – 95% confidence interval

Linear regression for the quantitative trait analysis of corneal curvature was undertaken for the 18 selected SNPs. There was no significant association for this trait with or without the inclusion of the covariates for age and gender.

## Discussion

Through analysis of the genome-wide significant SNPs originally identified as associated with AMD Fritsche et al. (2013, 23), we were able to confirm that 2 SNPs survived multiple testing in our KC analysis. These implicated the *ADAMTS9* and *TIMP3* loci as potentially playing a role in the pathogenesis of KC. We examined the trait of corneal curvature to explore the possibility that these associations were involved in this aspect of KC disease aetiology but there was limited evidence in their involvement through this mechanism.

The *ADAMTS9* (disintegrin and metalloproteinase with thrombospondin motifs 9) gene is a complex secreted enzyme that cleaves large aggregating proteoglycans including aggrecan and versican. It also has a protease-independent function in promoting the transport of a variety of secretory items from the endoplasmic reticulum to the Golgi apparatus. It is localized to chromosome 3p14.3-p14.2 and a similar region has been previously implicated in an Italian KC linkage study [25]. The gene is expressed in most eye tissues including the cornea (Ocular Tissue database (https://genome.uiowa.edu)). Other members of the *ADAMTS* family have previously been implicated in two prior linkage regions reported for KC with *ADAMTS7* identified at chromosome 15q22.33–24.2 and *ADAMTS18* at 16q22.3-q23.1 [26, 27]. Interestingly, in both cases, a protease gene was also present in the linked region although no KC causative gene has yet been identified from either of these linked regions.

*TIMP3* is a member of the *TIMP* (tissue inhibitors of metalloproteinases) family which represents a group of peptidases involved in the degradation of the ECM (extracellular matrix). Expression of this gene is induced in response to mitogenic stimulation and the protein is localized to the ECM. It is highly expressed in the cornea (Ocular Tissue database (https://genome.uiowa.edu)) and a previous study has indicated that differential gene expression showed a 14-fold decrease in expression of this gene in KC in cultured corneal stromal fibroblasts when comparing KC and non-KC controls [28]. In contrast, a previous study by De Bonis et al. did not find any specific mutations or novel variants in this gene in 302 Italian KC patients following the sequencing of its coding regions and they therefore ruled it out as being involved in KC [29]. Interestingly another member of the *TIMP* family (*TIMP1*) has also been implicated in KC where a significant reduction in both transcript level (*p* < 0.05) and protein (*p* < 0.0001) were reported [30]. Additionally, the SNP rs6609533 in *TIMP-1* was also reported as being associated with KC (OR 2.27, 95% CI, 1.06–4.76, *P* = 0.036) when comparing 140 KC patients and 150 healthy controls [31]. These findings suggest an increasing body of evidence for a role of TIMP genes in KC and more broadly speaking, the likely role of genes involved in the ECM as having some involvement in KC.

Our study also investigated the association of AMD-related genes with KC in females and males separately. Gender differences have been reported in KC, differing in terms of prevalence, clinical parameters, symptoms and treatment prognosis. Males present at a higher prevalence in most studies, and the present study showed the same trend, with 61.3% of the KC group being male [12, 13]. We also noted that the *TIMP3* gene appeared to show association only when males were considered whereas no sex-specific associations were noted with females. This therefore begs the question as to whether there are sex-specific genetic associations in KC which might therefore contribute to disease aetiology. However, this finding could also reflect that in the current study, there were more males than females and thus the significant associations observed in male cases could be related to this larger sample size.

In our analysis, we included a group of individuals without KC as our control group which was deliberately chosen to be older than the KC group to exclude or minimize the possibility of the occurrence of any KC. In undertaking our analysis, we included a co-variate of age in our analysis but the significant genetic associations for both the *TIMP3* and *ADAMTS9* genes disappeared. This questions whether the associations we have identified are true associations or perhaps represent allelic differences which might be age dependent. In a previous study of SNPs associated with AMD, we were able to show that different genotype frequencies were seen across different 10-year age groups for several SNPs (rs1061170 (*Y402H*), rs2274700, rs393955) in the complement factor H (*CFH*) gene, albeit at an older age. These different genotype frequencies occurred across the age range of 48–86 years where the prevalence of the low-risk homozygote rose with each increasing age group [32]. However, not all tested SNPs in that study showed an age-dependent change with SNP rs800292 in the *CFH* gene not being significant [32]. In addition, not all known AMD associated SNPs were examined for age differences in that study and thus there are no known reports of genotype frequencies varying with age for the majority of the currently assessed AMD SNPs.

As a further analysis in our current study, we grouped all KC cases and controls together and then split them into tertiles. The upper age tertile group (range 52 to 87 years) and lower tertile group (age range 5 to 34 years) consisted of 211 and 204 individuals separately and were compared for an age effect. Interestingly, for both rs6795735 and rs5749482, significant associations (*p* = 2 × 10^− 2^ and *p* = 2 × 10^− 3^, respectively) were identified between the two tertiles groups. While KC has typically been reported as developing at an early age, we did not expect to find an age effect in our KC study for the reported SNPs. The self-reported average age of KC onset typically ranges from 16.7 to 28.3 years, centering in the second or third decade of a patient’s life [6, 33–35]. However, the youngest-described KC patient in 2015 has been reported as only being four years old [36]. Furthermore, assessment of older KC patients has reported that the number of patients older than 50 years with KC is higher than before [37].

Power calculations for SNP rs6795735 (*ADAMTS9*) (minor allele frequency 0.52 in cases and 0.41 in controls) indicated 77% power to detect significant association when both genders were used. In the case of SNP rs5749482 (*TIMP3*) with a minor allele frequency of 0.19 in cases and 0.13 in controls, our study had 51.2% power to detect significant association when both genders were included. Subsequently, power would be expected to decrease if males and females were considered in separate analyses. However, in the case of SNP s5749482, power actually increased to 73% for a male only analysis but diminished to only 4.2% in a female only analysis. This reflected differences in allele frequencies between males and females for this SNP being 0.21 in KC cases and 0.10 in controls (males) but 0.16 in KC and 0.15 in controls (females). To obtain an appropriate sample size to be well powered at this SNP in females would require 17,136 KC cases and 25,704 controls. Clearly, this scenario would be highly unlikely to be achieved given the paucity of KC samples but does present an important limitation in assessment of gender differences when undertaking genetic associations. Given that KC is a relatively rare condition, the majority of DNA studies in KC typically have a small sample size (approximately 200 cases and 300 control subjects). Thus, the number of subjects in the present study are comparable with other studies but does raise an important issue in allele frequency differences that may exist between males and females as well as between individuals of different ethnicities. Another consideration in this study was age. While KC is not classified as an age-related disease due to its relatively early age of onset, there is a likelihood that older controls will be used as a comparison group to minimize the potential inclusion of incident KC. However, if a SNP exhibits differences in allele frequency with age then this must also be considered. Further studies are needed to resolve these issues, by performing genetic association tests between age-and gender matched groups to minimize any effects of age or sex. Ultimately, this will require very large samples sizes to address such issues which may be impossible to achieve in a relatively uncommon condition such as KC.

## Conclusions

KC is a complex condition where genetics appear to play a vital role [38, 39]. While many candidate genes and linkage regions have been identified in KC through previous twin/family and case-control studies, few genes have been consistently reported [17–20, 31, 40–86]. Given our previous observation of posterior segment changes in the eye of KC patients [21], we sought to assess whether genes implicated in the complex eye disease of AMD may also show some involvement in KC. While the data are suggestive, given that at least 2 SNPs survive multiple correction, their involvement in this disease is not confirmed as it appears that these associations can be influenced by age and gender co-variates. These observations may explain the lack of reproducibility in different KC studies depending on methodology but if it can be demonstrated that such associations do exist in larger or replicated cohorts then this would open new avenues for the involvement of genes involved in the KC disease pathway. Ultimately, the likely resolution to the answer of the suggested genetic associations described in this study as well as prior studies will most likely arise through undertaking a large scale GWAS for KC as this will identify genes in a hypothesis-free manner in a larger patient population.

## Supplementary information

**Additional file 1: Table S1.** Full names of genes studied in the present study. The table shows the full names of genes studied in the present study

## Data Availability

The datasets used and/or analysed during the current study are available from the corresponding author (Paul Baird) on reasonable request.

## Abbreviations

AMD: Age-related macular degeneration
CCT: Central corneal thickness
CI: Confidence intervals
CLEK: Collaborative Longitudinal Evaluation of Keratoconus
CXL: Corneal collagen cross-linking
DNA: Deoxyribonucleic acid
ECM: Extracellular matrix
GEM: GEnes in Myopia
GWAS: Genome wide association studies
HGF: Hepatocyte growth factor
KC: Keratoconus
MAF: Minor allele frequency
OCT: Optical coherence tomography
OR: Odd ratio
RVEEH: Royal Victorian Eye and Ear Hospital
SNPs: Single nucleotide polymorphisms
TIMP: Tissue inhibitors of metalloproteinases
