# Supplementary material for: Do age-related macular degeneration genes show association with keratoconus?
Source: Eye Vis (Lond). 2019 Dec 1;6:38. doi: 10.1186/s40662-019-0164-z (PMC6885317; doi:10.1186/s40662-019-0164-z)
Supplement: Supplementary file 1 — Additional file 1: Table S1. Full names of genes studied in the present study. The table shows the full names of genes studied in the present study [file 40662_2019_164_MOESM1_ESM.docx]

Additional file 1: Full names of genes studied in the present study. The table shows the full names of genes studied in the present study

Supplementary Table S1 Full names of genes studied in the present study

| **Name in full** | **Abbreviations** |
| --- | --- |
| Age-Related Maculopathy Susceptibility 2 | *ARMS2* |
| HtrA Serine Peptidase 1 | *HTRA1* |
| Complement Factor H | *CFH* |
| TNF Receptor Superfamily Member 10a | *TNFRSF10A* |
| Cholesteryl Ester Transfer Protein | *CETP* |
| Complement C3 | *C3* |
| Immediate Early Response 3 | *IER3* |
| Discoidin Domain Receptor Tyrosine Kinase 1 | *DDR1* |
| Transforming Growth Factor Beta Receptor 1 | *TGFBR1* |
| Collagen Type X Alpha 1 Chain | *COL10A1* |
| Complement C2 | *C2* |
| Complement Factor B | *CFB* |
| Apolipoprotein E | *APOE* |
| Complement Factor I | *CFI* |
| TIMP Metallopeptidase Inhibitor 3 | *TIMP3* |
| ADAM Metallopeptidase with Thrombospondin Type 1 Motif 9 | *ADAMTS9* |
| RAD51 Paralog B | *RAD51B* |
| Solute Carrier Family 16 Member 8 | *SLC16A8* |
| Lipase C, Hepatic Type | *LIPC* |
| Vascular Endothelial Growth Factor A | *VEGFA* |
| Collagen Type VIII Alpha 1 Chain | *COL8A1* |
| Filamin A Interacting Protein 1 Like | *FILIP1L* |

Reference

1. GeneCards®: The Human Gene Database [Internet]. Available from: https://www.genecards.org/Search/Keyword?queryString=FILIP1L.
